# Research on Function of Ribosomal Protein S6 Kinases, 1α and β, Based on Molecular Cloning and siRNA-Based Interference in Juvenile Blunt Snout Bream (*Megalobrama amblycephala*)

**DOI:** 10.3390/biology13110875

**Published:** 2024-10-28

**Authors:** Jiaze Gu, Haifeng Mi, Mingchun Ren, Dongyu Huang, Ahmed Mohamed Aboseif, Hualiang Liang, Lu Zhang

**Affiliations:** 1 Wuxi Fisheries College, Nanjing Agricultural University, Wuxi 214081, China; 2 Tongwei Agricultural Development Co., Ltd., Key Laboratory of Nutrition and Healthy Culture of Aquatic, Livestock and Poultry, Ministry of Agriculture and Rural Affairs, Healthy Aquaculture Key Laboratory of Sichuan Province, Chengdu 610093, China; 3 Key Laboratory of Integrated Rice-Fish Farming Ecology, Ministry of Agriculture and Rural Affairs, Freshwater Fisheries Research Center, Chinese Academy of Fishery Sciences, Wuxi 214081, China; 4 National Institute of Oceanography and Fisheries (NIOF), Academy of Scientific Research and Technology (ASRT), Cairo 11796, Egypt

**Keywords:** molecular cloning, juvenile blunt snout bream, ribosomal protein S6 Kinase 1, RNA interference

## Abstract

The aim of this study was to investigate the effects of S6K1α and β on the expression of glycolysis- and gluconeogenesis-related genes in juvenile blunt snout bream. Two isoforms, α and β, of ribosomal protein S6 kinase 1 in blunt snout bream were cloned and characterized, and their expression patterns were examined in vivo. The sequence analysis showed that *s6k1α* and *s6k1β* contain open reading frames of 2217 and 1497 bp, encoding 738 and 498 amino acids, respectively. Both S6K1α and S6K1β consist of an S_TKc domain and an extended S_TK_X domain. *s6k1α* and *s6k1β* were abundantly expressed in the heart and gonads. siRNAs were designed, and the experiment showed that α-siRNA inhibited *s6k1α* and *s6k1β* expression, but β-siRNA exclusively inhibited *s6k1α* expression (*p* < 0.05). α-siRNA upregulated the expression levels of *gk* and *pk*, while β-siRNA upregulated *pepck* and *g6p* expression (*p* < 0.05). The expression of *g6pdh* was found to be downregulated, but the *gs* mRNA level was overexpressed after treatment with α-siRNA and β-siRNA (*p* < 0.05). In the present experiment, S6K1α was more intimately involved in the regulation of gluconeogenesis when only S6K1α was inhibited, whereas the inhibition of both S6K1α and S6K1β collectively co-regulated glycolysis.

## Simple Summary

The aim of this study was to investigate the effects of S6K1α and β on the expression of glycolysis- and gluconeogenesis-related genes in juvenile blunt snout bream. The two isoforms, α and β, of S6K1 in blunt snout bream were successfully cloned and characterized, and their expression patterns were examined in vivo. In this study, we designed multiple sets of siRNAs to specifically degrade *s6k1α* and *s6k1β* expression in this fish. α-siRNA inhibited both *s6k1α* and *s6k1β* expression, but β-siRNA exclusively inhibited *s6k1α* expression. S6K1α was more intimately involved in the regulation of gluconeogenesis when only S6K1α was inhibited, whereas the inhibition of both S6K1α and S6K1β collectively co-regulated glycolysis.

## 1. Introduction

Phosphorylation of protein molecules is a crucial regulatory mechanism in intracellular signal transduction, and the involvement of protein kinases is indispensable for almost all intracellular activities [1]. Rapamycin-targeting protein (mTOR) regulates mRNA translation and protein synthesis by phosphorylating ribosomal protein S6 kinase 1 (S6K1) and eukaryotic translation initiation factor 4E binding protein (4E-BP) [2]. mTORC1 phosphorylates and activates two isoforms of S6 kinase, namely, S6K1 and S6K2 [3]. Ribosomal protein S6 kinases are predominantly localized in the cytoplasm and are essential for growth factor-regulated cell proliferation, pathways involving cell motility such as metastases, immune response, and tissue repair [4,5]. S6K1 plays an important role downstream of rapamycin-targeting protein (mTOR) activated by mTOR-dependent phosphorylation, and it is the best-characterized effector that mediates the increase in protein synthesis and cell proliferation [6]. Furthermore, S6K1 is involved in the feedback regulation of mTORC2 and mTORC1, mediating cell survival and regulating insulin resistance [6]. There are two isoforms of S6K1, which is considered a key enzyme in the activation of protein synthesis through mitogenic signals [7]. The two isoenzymes may have different functions within the cell due to differences in phosphorylation sites and nuclear localization signal locations, and they are usually distinguished as α and β [8].

Previous studies have demonstrated that mTORC1 not only directly affects the growth and proliferation of pancreatic β-cells [9], but S6K can improve glucose tolerance by increasing insulin secretion without increasing the number of pancreatic β-cells, suggesting that mTORC1 regulates insulin secretion mainly through the S6K pathway [10]. Furthermore, the injection of adenoviruses in the mouse hypothalamus to overexpress p70S6k1 resulted in increased gluconeogenesis [11]. Meanwhile, S6K knockout mice were protected against diet-induced insulin resistance [12]. However, the administration of rapamycin, a specific inhibitor of mTOR, has different effects on humans. Although rapamycin partially inhibits mTOR-mediated S6K phosphorylation, it stimulates insulin-mediated glucose uptake in vivo under conditions that activate the mTOR/S6K pathway [13]. Nevertheless, experimental animals may exhibit different effects on glucose metabolism, and there is increasing evidence for the regulation of glucose metabolism by ribosomal protein S6 kinases [14,15].

Blunt snout bream (*Megalobrama amblycephala*) is widely cultured worldwide as a herbivorous freshwater fish [16]. As is the case with other fish species, poor carbohydrate utilization leads to adverse effects such as reduced growth performance and liver diseases [17]. Therefore, there has been considerable interest in enhancing the carbohydrate utilization of fish. Liang et al. found that both 2.70% dietary arginine and 2.94% dietary leucine levels activated the overexpression of *s6k1*, leading to the upregulation of gluconeogenesis by increasing *pepck* and *g6p* mRNA levels, which elevated the plasma glucose content in juvenile blunt snout bream [18,19]. However, high levels of amino acids also promoted TOR activity, which contributed to insulin resistance through various mechanisms aside from the S6K signaling pathway [12].

Previous studies have mainly focused on the effects of glucose metabolism due to the activation of S6K kinase by exogenous nutrients or other upstream signaling pathways. However, there is a lack of research on the inhibition of S6K signaling in aquatic animals. In particular, there is no research on the function of S6K in glycolysis and gluconeogenesis in blunt snout bream. Therefore, in the present study, we cloned the full-length cDNA sequences of the two S6K1 kinase isoforms, α and β, while tissue distribution and expression were measured separately. In addition, the relevant effects on glycolysis- and gluconeogenesis-related genes were explored via inhibition of S6K kinase. The data obtained in this study provide new insights into the function and role of S6K1 in the regulation of glycolysis and gluconeogenesis in blunt snout bream.

## 2. Materials and Methods

### 2.1. Fish Management and Sample Collection

Experimental blunt snout breams (average weight: 10 g) were obtained from Freshwater Fisheries Research Centre (FFRC) of the Chinese Academy of Fishery Sciences (Wuxi, China) and cultured. Prior to the start of the experiment, the fish were acclimatized in temporary 2 m × 2 m × 2 m cages. The fish were fed a commercial die twice daily to satiety (08:00 and 17:00). The culture water’s oxygen content, temperature, and photoperiod were consistent with the conditions used in our previous experiments [20].

The experimental fish were anaesthetized by MS-222 (100 mg/L) after 24 h of starvation. For subsequent gene cloning and tissue distribution, fish samples, including the kidney, liver, gill, brain, muscle, cholecyst, foregut, midgut, hindgut, heart, and gonads, were collected and quickly dissected; they were then frozen in liquid nitrogen and stored at −80 °C until use.

### 2.2. Identification and cDNA Cloning

We genetically annotated and searched for *s6k1α* and *s6k1β* through previous transcriptional sequencing of blunt snout bream. After near-relative sequences were aligned for subsequent gene cloning, the RACE method and sequencing techniques were conducted by Bio-Transduction Lab Co., Ltd. (Wuhan, China). RNA was extracted from the tissues using TRIzol (Takara), and the quality was tested. The RevertAid First Strand cDNA Synthesis Kit was used for cDNA first-strand synthesis (Fermentas). The open reading frame (ORF) full-length amplification system was as follows: PCR-grade water at 15.0 μL, 2× PCR buffer for KOD FX Neo at 25.0 μL, dNTP Mix (10 mM) at 1.0 μL, KOD FX Neo (1 U/μL) at 1.0 μL, cDNA first strand at 5.0 μL, primer F (10X) at 1.5 μL, and primer R (10X) at 1.5 μL. A 1% agarose gel was prepared for electrophoresis to verify the length of the primer products, and the correct band was cut for recovery. T4 ligase kits (Takara) were used to ligate the PCR recovery products overnight at 4 °C, which were then transformed into *E. coli* receptor cells. After the identification and screening of positive clones, sequencing was performed to obtain full-length cDNA sequences. Specific primers were designed for ORF full-length amplification, and CDS clones are shown in Table 1.

### 2.3. Sequence Characterization and Analysis

The open reading frames of the full-length sequences were analyzed using NCBI Open Reading Frame Finder (https://www.ncbi.nlm.nih.gov/orffinder/ (accessed on 10 November 2023)). The molecular properties of S6K1α and S6K1β, including their amino acid sequence/atomic composition, calculated as molecular mass, pI, extinction coefficient, estimated half-life, instability index, and aliphatic index, were predicted using the ExPASy tool (https://www.expasy.org/ (accessed on 12 November 2023)). The sequenced amino acid’s secondary structure was predicted using the Prabi-Gerland site (https://npsa-prabi.ibcp.fr/cgi-bin/npsa_automat.pl?page=npsa_sopma.html (accessed on 12 November 2023)). The prediction of protein structure and function at the CDS region was performed with the SMART tool (http://smart.embl-heidelberg.de/ (accessed on 15 November 2023)). The prediction of the 3D stereo model of S6k protein was conducted using the SWISS MODEL tool (http://swissmodel.expasy.org/ (accessed on 15 November 2023)). Signal peptides and transmembrane regions were predicted using SignalIP (http://www.cbs.dtu.dk/services/SignalP/ (accessed on 18 November 2023)) and TMHMM SerVer 2.0 (http://www.cbs.dtu.dk/services/TMHMM/ (accessed on 18 November 2023)), respectively. The S6K protein sequences were obtained from the NCBI database (https://www.ncbi.nlm.nih.gov/ (accessed on 20 November 2023)). DNAMAN and MegAlign software were used for multiple sequence alignment. The Clustal-W multiple alignment algorithm was used to assess the percentage of amino acid conservation. The phylogenetic tree was constructed using MEGA-X software via the neighbor-joining (NJ) method with 10,000 bootstrap replicates.

### 2.4. Knockdown of S6K1 in Liver

A knockdown method was selected for silencing interfering genes using small interfering RNA (siRNA). The design and production of the siRNA (target gene and negative control) were carried out by Genepharma (Shanghai, China). Three pairs of primers were designed separately for each of the functional structural domains of the two different isoforms for the experiment. The sequences of the siRNA interference fragments for *s6k1α* and *s6k1β* are shown in Table 1. In vivo transfection of the animal RNA was performed using Entranster^TM^-in vivo reagent from Engreen Biosystem Co., Ltd. (Beijing, China). Nucleic acids (μg) and EntransterTM-in vivo reagent (μL) were mixed in a 2:1 ratio. The nucleic acids were dissolved in sterile DEPC water. A total of 100 μL of transfection complexes, whose osmotic balance was ensured by adding a final concentration of 0.9% saline in the base solution, was injected intraperitoneally into the experimental juvenile blunt snout bream at a dosing concentration of 5 mg/kg. The fish in the negative control condition were injected with a single injection of an equal volume of 100 μL of transfection complexes at a final concentration of 0.9% saline. After 12 h of gene silencing, the liver was collected and frozen in liquid nitrogen and stored at −80 °C until use.

### 2.5. Gene Expression and qRT-PCR

The RNAiso Plus kit (Takara) was used to treat different kinds of tissues for RNA extraction. A NanoDrop 2000 spectrophotometer (Shanghai, China) was used to test and quantify the concentration of RNA. The quantitative reverse transcription polymerase chain reaction (qRT-PCR) analysis was conducted using a 7500 Real-Time PCR System (Applied Biosystems Inc. (ABI), Foster City, CA, USA). The relative gene expression was measured using the relative standard curve (RSC) method. The amount of the target and internal reference (*β-actin*) genes was determined using the standard curves. Subsequently, the internal reference genes were used to normalize the gene expression in the experimental and control conditions. Four biological replicates were used in the qRT-PCR. Specific primers were designed using Primer Premier 6.0 software for the real-time PCR system (Table 1).

### 2.6. Biostatistical Analysis

All data were analyzed using IBM SPSS v. 26.0 software. The data were analyzed for significant differences by means of independent t-tests and one-way ANOVA. All results are presented as means ± SEM in the graphs, labeled with different letters to indicate significant differences between groups (*p* < 0.05).

## 3. Results

### 3.1. Molecular Characterization of s6k1α and s6k1β

**Figure 1:**
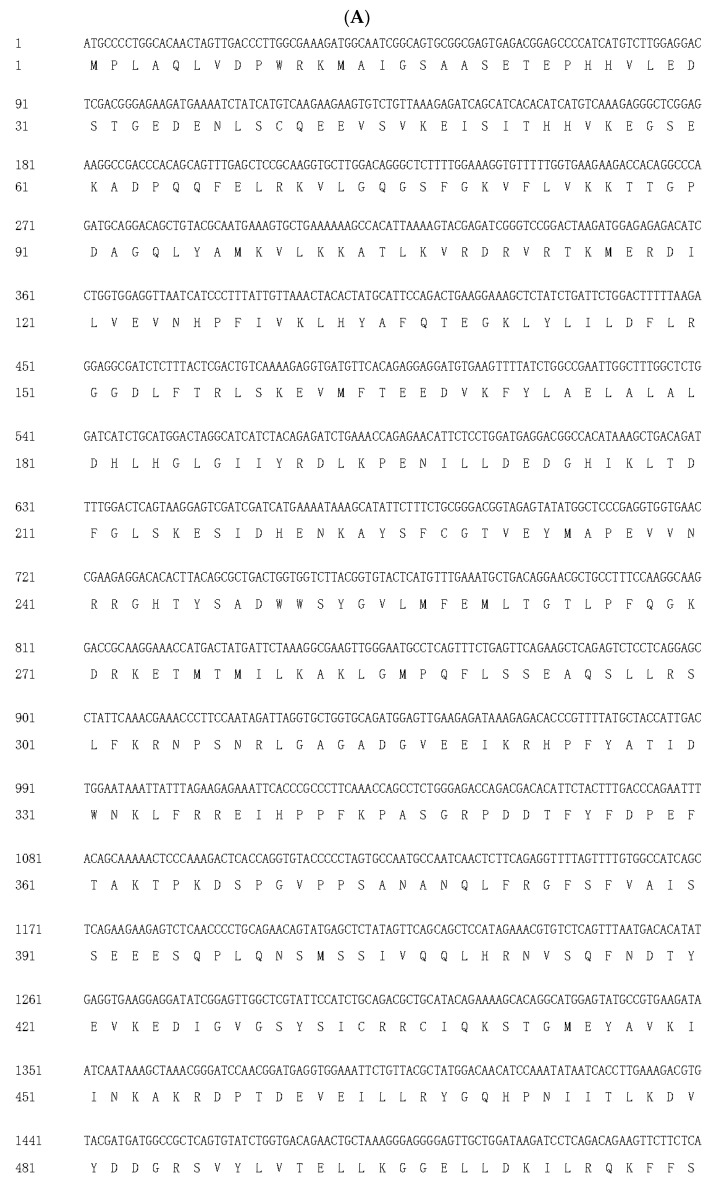

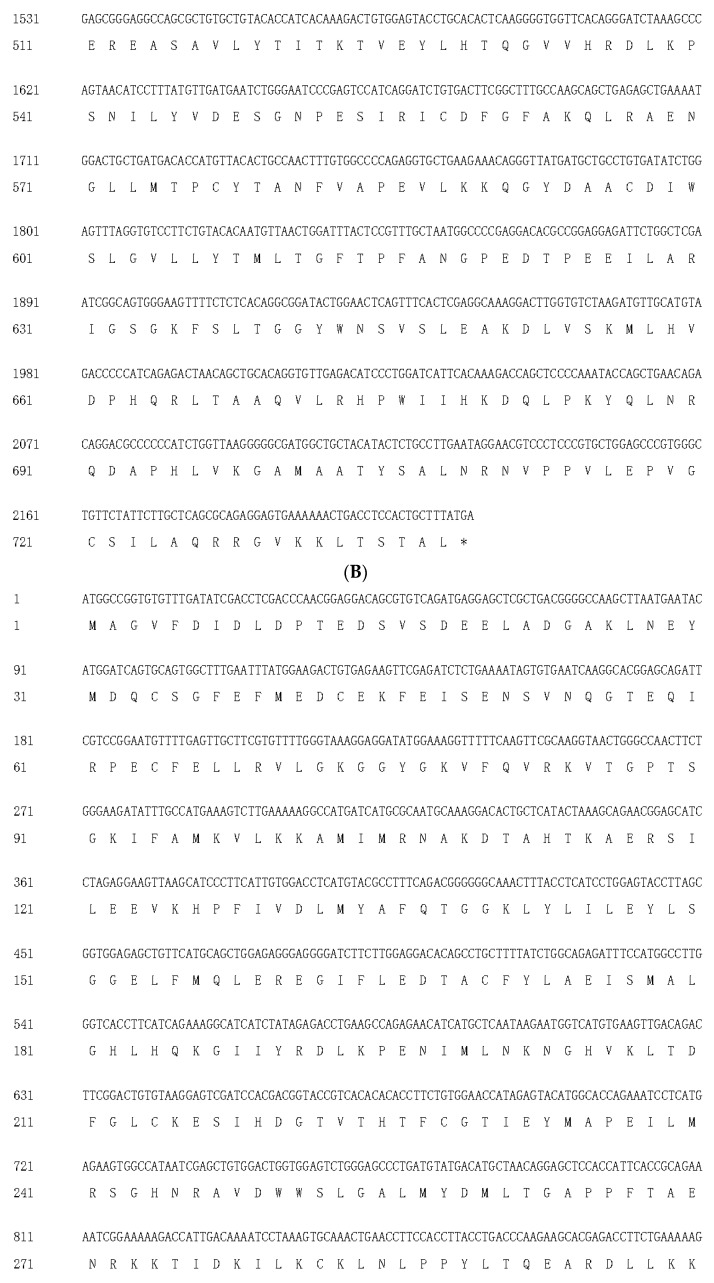

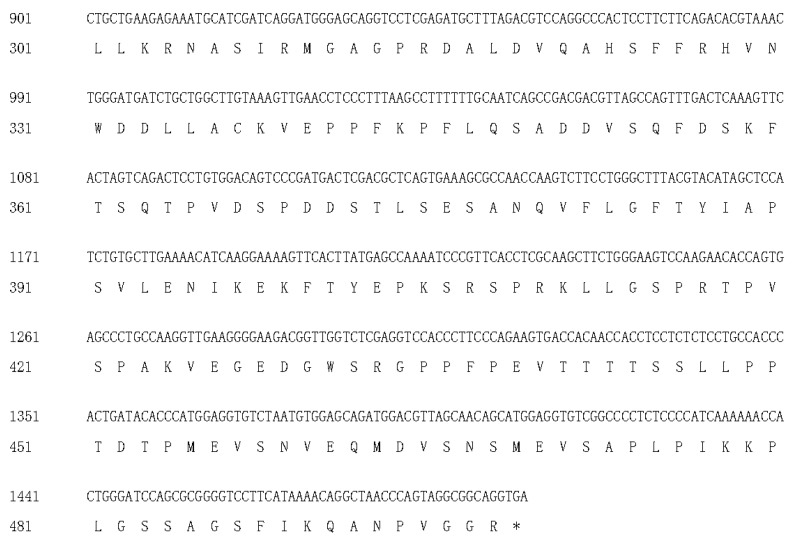
The CDS and deduced amino acid sequences of S6K1α and S6K1β. (**A**) S6K1α; (**B**) S6K1β. The cDNA sequences of s6k1α (accession number: OR896619.1) and s6k1β (accession number: OR896620.1) have been submitted to the GenBank nucleotide sequence database.

**Figure 2:**
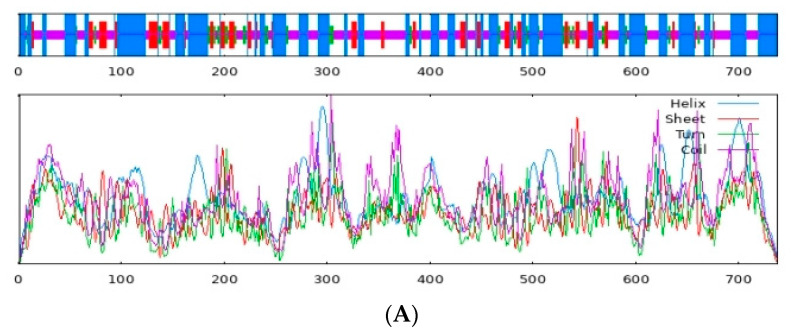

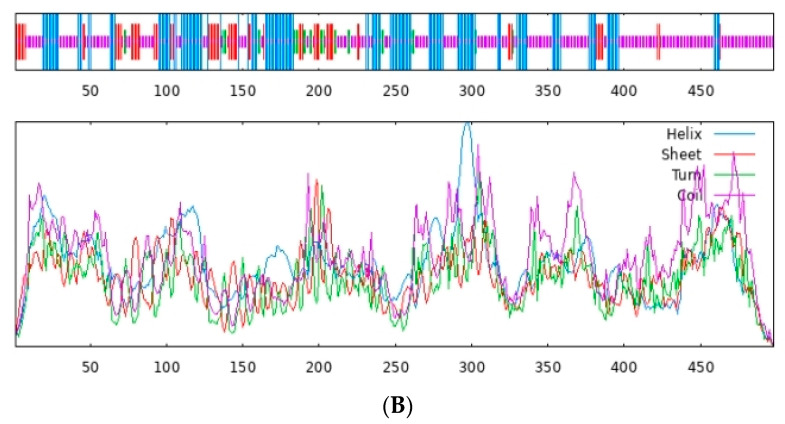
Secondary structure predictions of S6K1α and S6K1β. (**A**) S6K1α is predicted to contain 41.73% α-helix, 12.87% extended strand, 7.59% β-turn, and 37.80% random coil. (**B**) S6K1β is predicted to contain 29.92% α-helix, 12.85% extended strand, 5.42% β-turn, and 51.81% random coil. Color blue means α-helix; color red means extended strand; color green means β-turn; color purple means random coil. Numbers identify the location of amino acids.

The cDNA sequences of s6k1α (accession number: OR896619.1) and s6k1β (accession number: OR896620.1) have been submitted to the GenBank nucleotide sequence database. The full-length cDNA sequence of the s6k1α gene is 3824 bp, which contains a complete 2217 bp open reading frame encoding 738 amino acids (from 329 bp to 2545 bp) (Figure 1A). S6k1β is 3242 bp in size and contains a complete open reading frame of 1497 bp, encoding 498 amino acids (from 118 bp to 1614 bp) (Figure 1B). The results of the basic sequence analyses, including molecular formula, weight, theoretical pI, extinction coefficient, instability index, estimated half-life, aliphatic index, and grand average of hydropathicity (GRAVY), are shown in Table 2. The prediction of the S6K1α secondary structure indicated 41.73% α-helices, 12.87% extended strands, 7.59% β-turns, and 37.80% random coils (Figure 2A). The prediction of the S6K1β secondary structure showed 29.92% α-helices, 12.85% extended strands, 5.42% β-turns, and 51.81% random coils (Figure 2B).

**Figure 3:**
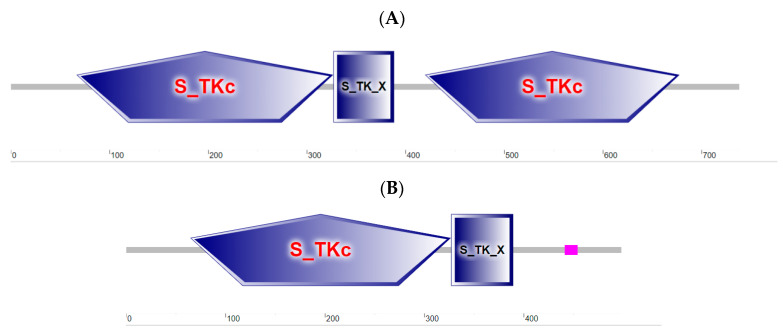
Prediction of protein structure and function by SMART of S6K1α and S6K1β. (**A**) S6K1α. Consists of two S_TKc domains (located at bits 67 to 326, 420 to 677) and one S_TK_X domain (located at bits 327 to 388). (**B**) S6K1β. Consists of one S_TKc domain (located at bits 65 to 326) and one S_TK_X domain (located at bits 327 to 389). The pink square consists of a low complexity domain (located at bits 441 to 454).

The prediction of protein structure and function via SMART found that both S6K1α and S6K1β consist of a serine/threonine protein kinase catalytic structural domain (S_TKc) and an extended serine/threonine protein kinase domain (S_TK_X) (Figure 3).

### 3.2. Sequence Alignment and Phylogenetic Analysis of S6k1α and S6k1β

**Figure 4:**
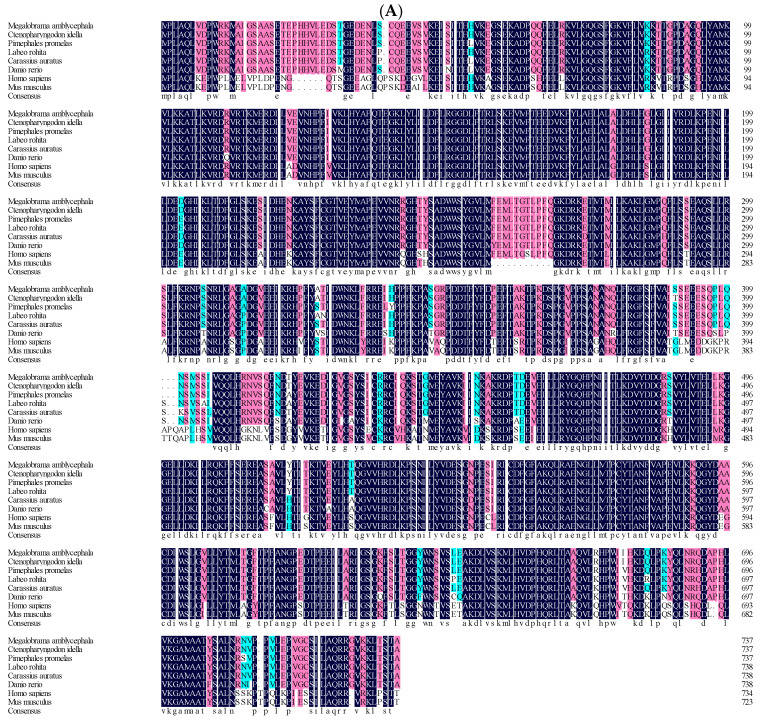

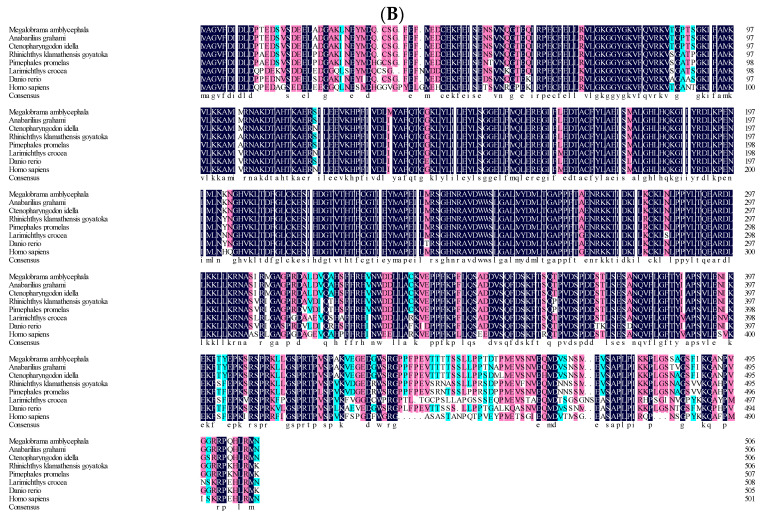
Multiple sequence alignments of S6K1α and S6K1β amino acid sequences between *Megalobrama amblycephala* and other species. The numbers on the right side of the sequence indicate the position of the amino acid residues. Identical residues are indicated with white letters on a black background; similar residues with an identity above 75%, identity above 50%, and identity below 50% are indicated in pink, blue, and white, respectively. (**A**) S6K1α. The species used were *Megalobrama amblycephala* (GenBank accession no. WPQ74743.1), *Ctenopharyngodon idella* (GenBank accession no. XP_051737588.1), *Pimephales promelas* (GenBank accession no. XP_039540962.1), *Labeo rohita* (GenBank accession no. XP_050954136.1), *Carassius auratus* (GenBank accession no. XP_026093793.1), *Danio rerio* (GenBank accession no. XP_009295647.1), *Homo sapiens* (GenBank accession no. NP_002944.2), *Mus musculus* (GenBank accession no. AAA50300.1). (**B**) S6K1β. The species used were *Megalobrama amblycephala* (GenBank accession no. WPQ74744.1), *Anabarilius grahami* (GenBank accession no. ROL54336.1), *Ctenopharyngodon idella* (GenBank accession no. XP_051765238.1), *Rhinichthys klamathensis goyatoka* (GenBank accession no. XP_056103607.1), *Pimephales promelas* (GenBank accession no. XP_039533993.1), *Larimichthys crocea* (GenBank accession no. XP_010747297.3), *Danio rerio* (GenBank accession no. XP_021327969.1), *Homo sapiens* (GenBank accession no. NP_001258989.1).

**Figure 5:**
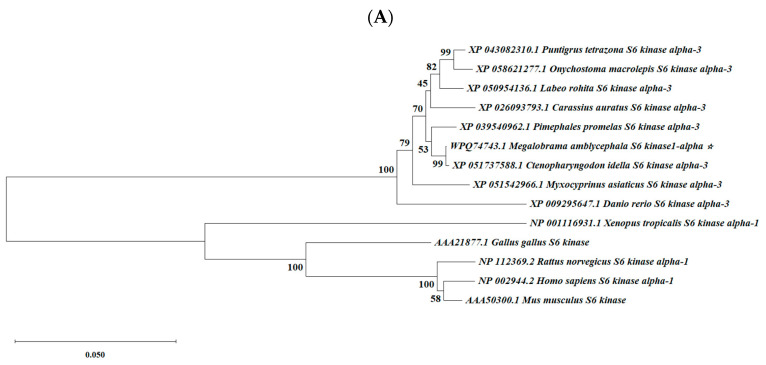

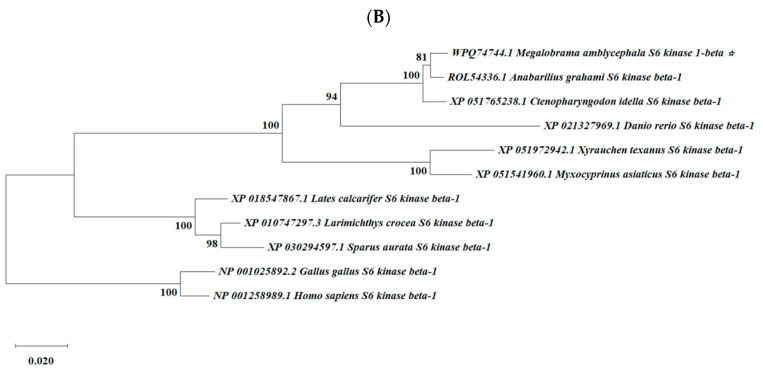
Phylogenetic tree based on S6K1α and S6K1β sequences from *Megalobrama amblycephala* and other species using the neighbor-joining (NJ) method. (**A**) S6K1α; (**B**) S6K1β. The NJ tree was constructed with MEGA X with 10,000 bootstrap replicates. The Genbank accession numbers from related organisms are listed next to each species. The numbers next to the branches represent the bootstrap support values.

Figure 4 shows the results of the multi-species amino acid sequence alignment, indicating that both S6K1α and S6K1β exhibit a relatively high degree of conservatism. The alignment revealed that the S6K1α of *M. amblycephala* exhibited a higher sequence identity with that of *Ctenopharyngodon idella* (GenBank accession no. XP_051737588.1) and *Pimephales promelas* (GenBank accession no. XP_039540962.1) than the other fishes, showing 99.9% and 98.8% species similarity, respectively (Table 3). The neighbor-joining trees constructed from the multi-species amino acid sequences indicate that the S6K1α of *M. amblycephala* is closer to that of other fish species and more distant from mammals, birds and amphibians (Figure 5A). In comparison, the S6K1β of *M. amblycephala* is more closely related to that of *Anabarilius grahami* (GenBank accession no. ROL54336.1) and *Ctenopharyngodon idella* (GenBank accession no. XP_051765238.1) (Table 4). According to the phylogenetic tree, *M. amblycephala* S6K1β is classified in the teleost S6K beta clade, which is most closely related to the carp family *Cyprinidae*, followed by the teleost classified in the order *Perciformes*, and it is distantly related to mammals and birds (Figure 5B). Similarly, the predicted serine/threonine protein kinase catalytic structural domains of S6K1α and S6K1β in *M. amblycephala* show essentially identical nucleotide and amino acid sequences to non-fish species, suggesting that the two genes are phylogenetically conserved, a finding that has also been verified in several vertebrates.

### 3.3. Tissue Distribution of s6k1α and s6k1β mRNA in Megalobrama amblycephala

**Figure 6:**
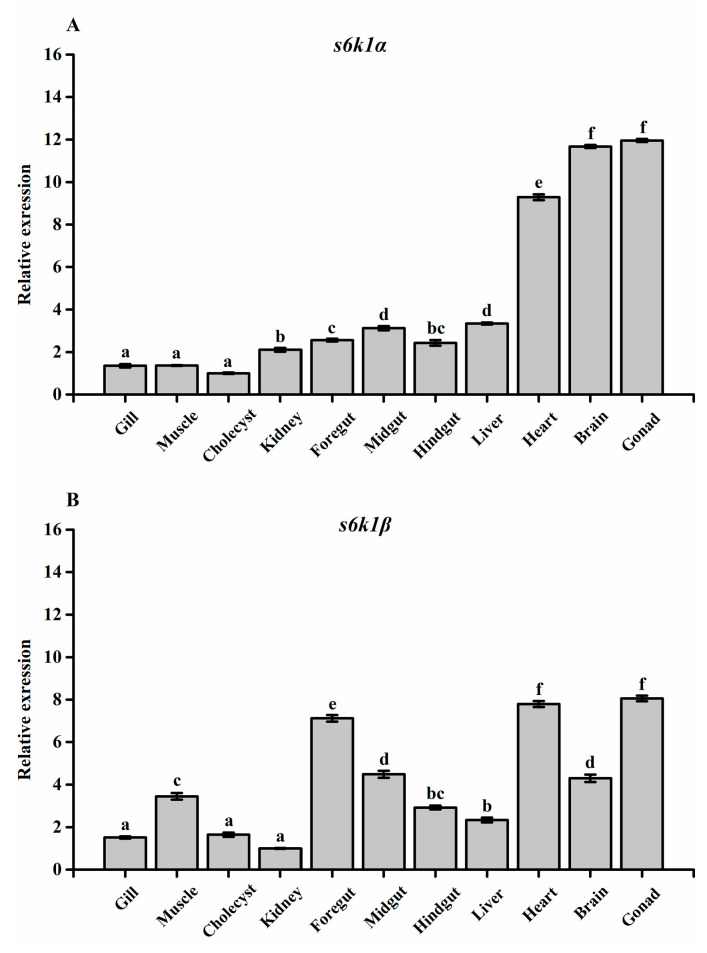
Tissue distribution of *s6k1α* and *s6k1β* mRNA in *Megalobrama amblycephala.* (**A**) *s6k1α;* (**B**) *s6k1β*. The results of the data are presented as mean ± SEM (n = 4). Columns with different letters represent significant differences between groups (*p* < 0.05).

The spatial distribution of *s6k1α* and *s6k1β* in tissues is shown in Figure 6. Both *s6k1α* and *s6k1β* were detected in all sampled tissues. In these tissues, *s6k1α* was more abundantly expressed in the heart, brain, and gonads (*p* < 0.05), while *s6k1β* was most highly expressed in the foregut, heart, and gonads (*p* < 0.05).

### 3.4. Knockdown of s6k1α and s6k1β by siRNA in Megalobrama amblycephala

**Figure 7:**
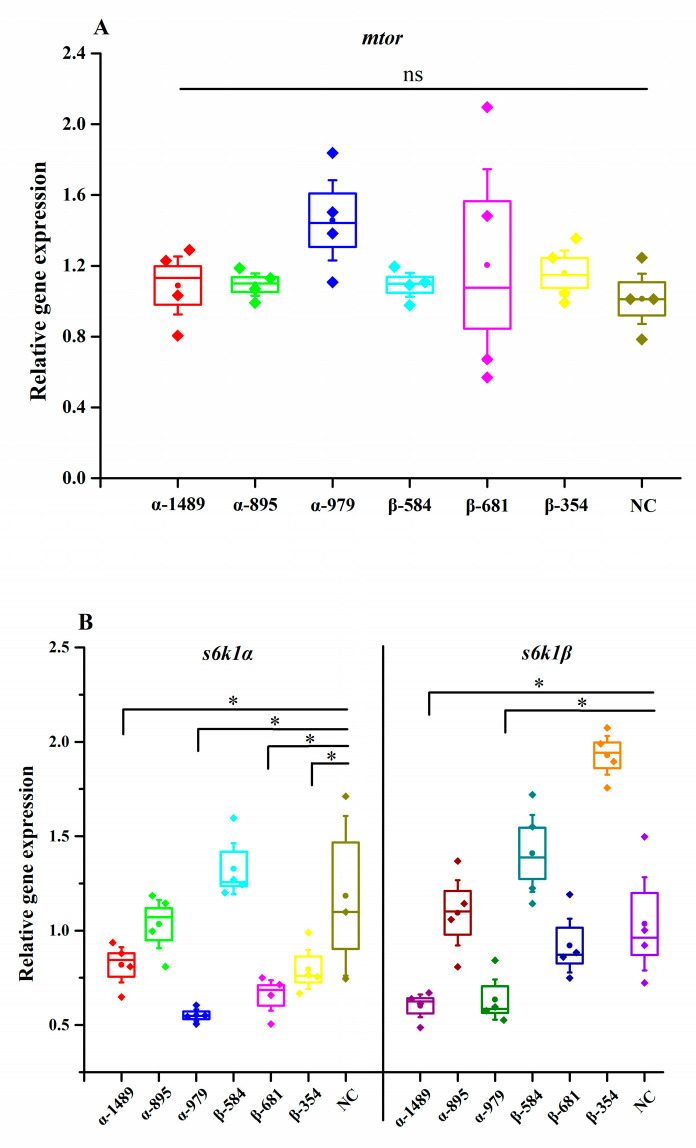
Knockdown of *s6k1α* and *s6k1β* by siRNA in *Megalobrama amblycephala.* (**A**) *mtor;* (**B**) *s6k1α* and *s6k1β*. The results of the data are presented as mean ± SEM (n = 4). Horizontal line markings indicate significance between data: ns means no significance; * means significance (*p* < 0.05).

The results of the treatment with siRNA are shown in Figure 7. No significant alterations were observed in the levels of *mtor* mRNA expression (*p* > 0.05). Both α-1489 and α-979 siRNA treatments significantly inhibited the expression of *s6k1α* and *s6k1β* in the fish (*p* < 0.05). However, the treatments with β-681 and β-354 siRNA demonstrated a notable inhibitory impact on *s6k1α* expression only (*p* < 0.05).

### 3.5. Effects of s6k1α and s6k1β siRNA on Glycolysis- and Gluconeogenesis-Related Genes

**Figure 8:**
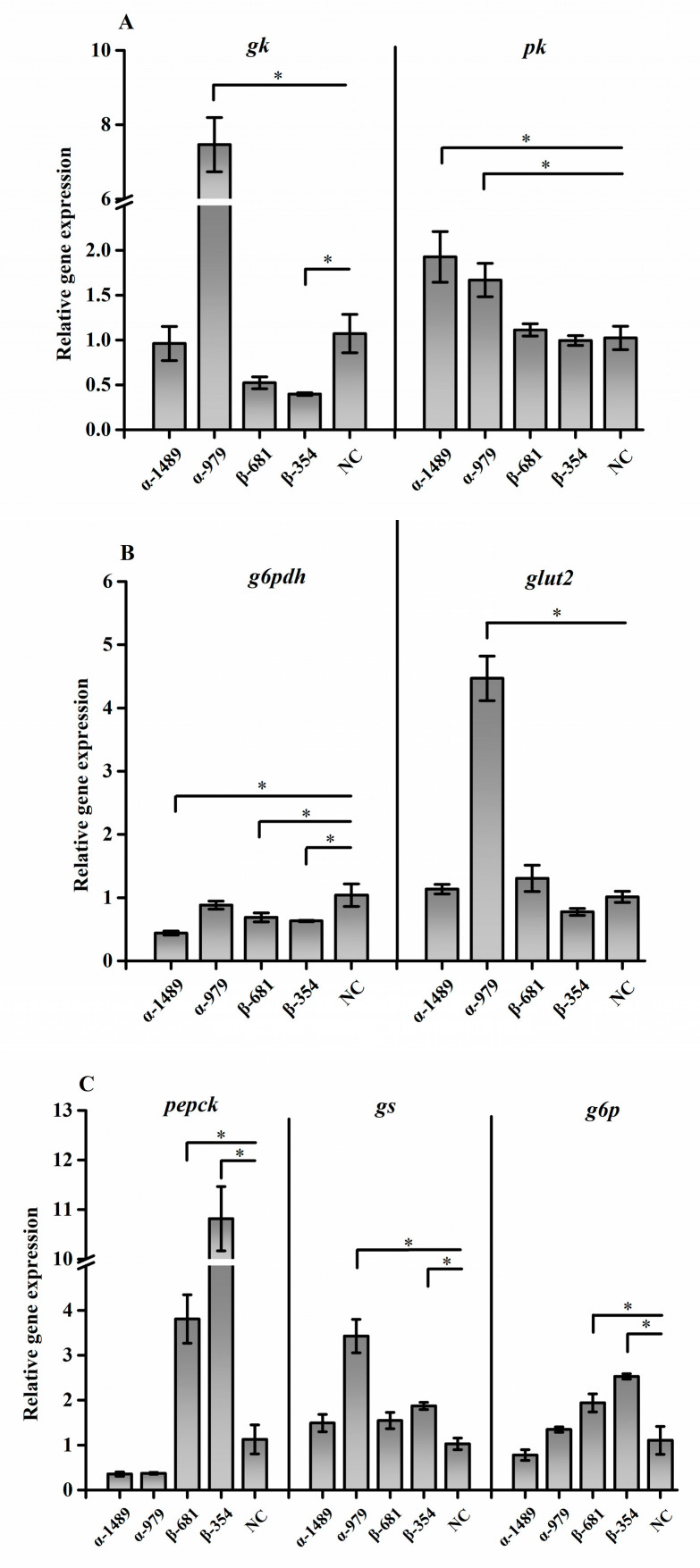
Effects of *s6k1α* and *s6k1β* on glucose metabolism in *Megalobrama amblycephala.* (**A**) *gk*, *pk;* (**B**) *g6pdh*, *glut2*; (**C**) *pepck*, *gs*, *g6p*. The results of the data are presented as mean ± SEM (n = 4). Horizontal line markings indicate significance between data: * means significance (*p* < 0.05).

The expression of genes related to glucose metabolism after siRNA treatment is presented in Figure 8. The expression of *pk* mRNA was significantly upregulated in the α-1489 and α-979 treatment groups (*p* < 0.05). The relative expression of *gk* mRNA reached a maximum for the α-979 treatment group and a minimum for the β-354 treatment group (*p* < 0.05). The expression of *g6pdh* was found to be downregulated in the α-1489, β-354, and β-681 treatment groups compared to the control group (*p* < 0.05). The expression of *glut2* was elevated in the α-979 treatment group (*p* < 0.05). Both β-354 and β-681 significantly upregulated the expression of *pepck* and *g6p* (*p* < 0.05). β-354 and α-979 upregulated the level of *gs* (*p* < 0.05).

## 4. Discussion

### 4.1. Molecular Characterization, Sequence Alignment, and Phylogenetic Analysis of s6k1α and s6k1β

Helical and folded structures are efficient for tightly stacking atoms on proteins, thus keeping the proteins highly compacted. The helical configuration is the most compact secondary structure. Both S6K1α and S6K1β were found to contain a relatively high proportion of helical structures, which allows for a greater variety of amino acids supported by the secondary structure and their relative stability [21]. As an effector of TOR substrates, the functional structure of S6K inevitably intersects with that of TOR, as does the MAPK family, which can also have an activating function. The S_TKc domain is also featured on p38MAPK of the remaining family of MAPKs and the TOR protein in *Bombyx mori* and other non-fish species [22,23,24,25,26].

The classification of ribosomal protein S6 kinases has not been clearly defined in current research. Consequently, the majority of these kinases are distinguished by their different subcellular localizations and protein molecular weights, including P70S6K and P85S6K (abbreviated as S6K1 and S6K2, respectively), and some are designated as α and β for differentiation purposes [7,11,27]. From an evolutionary perspective, because of the teleost-specific whole-genome duplication (WGD) and ploidy that occurred hundreds of millions of years ago, multiple subtypes of S6Ks are constantly being discovered in fish [28,29]. The evolutionary tree indicated that the sequence identity of *M. amblycephala* S6K1α is strikingly similar to that of S6Kα-3 in aquatic fish. With respect to the non-fish species’ S6K1α or S6Kα-1 and S6K1β, the nomenclature is more homogeneous and consistent. Therefore, renaming this gene to S6Kα-3 would be more accurate and consistent with the mainstream definition, while the other S6K isoforms still need to be further explored in *M. amblycephala*. In particular, a variety of factors, such as brackish water quality, food source, and external stresses in the fish environment, can affect fish gut health and gut microorganisms, which may be the reason for the further differentiation of S6K, which has long been recognized for its important protein metabolism function in fish [30,31]. This could also indicate that the gene duplication of S6K1 or S6Kα is more abundant in aquatic fish compared to other non-fish species. Thus, although both isoforms are highly conserved and more similar to grass carp in the *Cyprinidae* family, S6K1β, rather than S6K1α, is more likely to be retained as a repetitive sequence in the genome of blunt snout bream.

### 4.2. Tissue Distribution of s6k1α and s6k1β mRNA in Megalobrama amblycephala

Although the S6K gene is ubiquitously expressed in human adult tissues and other species [32,33], less is known about its expression patterns in fish. Similarly to the observations in cashmere goat (*Capra hircus*), S6K had high expression in the heart tissue [34]. In addition, the *BdS6K* gene was more highly expressed in the eggs of the oriental fruit fly (*Bactrocera dorsalis*, Hendel) and in the ovary of *Ae. aegypti* (*AeS6K*) [23,35]. As is the case with the majority of species, the elevated expression of the *s6k* gene in the gonads of blunt snout bream may be attributed to the need for increased S6K signaling to mediate access to nutrients for the accumulation of ribosomal proteins during gonadal development [36,37].

### 4.3. Knockdown of s6k1α and s6k1β by siRNA and Effects on Glycolysis- and Gluconeogenesis-Related Genes in Megalobrama amblycephala

In this study, mTOR was not activated following siRNA treatment, indicating that the design of the siRNA primers was effective in specifically inhibiting the expression of the downstream substrate S6K. In contrast to previous studies, this is the first study in which the upstream mTOR blocker, rapamycin, was not employed as a means of achieving S6K degradation. The two different siRNA primers exhibited a specific inhibitory effect, with the α isoform demonstrating a somewhat broader inhibitory capacity than the β isoform, which demonstrated a selective inhibitory effect on S6K1a only, while leaving S6K1β unaltered. From an observation of the evolutionary tree, it is also clear that the different species exhibit a single β-subtype. The results of the present experiment also support the hypothesis that the α isoform of the S6K1 protein plays a primary physiological role in *Megalobrama amblycephala*.

In recent years, researchers have discovered that targeting the rapamycin (TOR)/protein S6 kinase 1 (S6K1) signaling pathway via interferential treatment with exogenous nutrients, such as amino acids and drugs, could regulate glucose metabolism through a negative feedback mechanism [18,38,39]. The targeted regulation of S6K phosphorylation independently of mTOR has demonstrated potential as a means of regulating glucose metabolism [40]. The presence of the glucose transporter GLUT2 allows for bidirectional mediation of glucose transport into hepatocytes [41,42]. In this experiment, *glut2* mRNA expression was significantly upregulated after treatment with α-979 to promote glucose entry into the liver. However, as *gk* and *pk* are two crucial rate-limiting enzymes for glycolysis in the gluconeogenic pathway [43], both are overexpressed after the α-siRNA treatment. In the present study, S6K1s, which were inhibited by α-siRNA, were observed to activate glycolytic metabolism de novo by promoting *gk* expression. Moreover, PK, as a reaction in the final step of glycolysis [19], exhibits a similar pattern to that of *gk*, indicating its involvement in and promotion of the entire glycolytic process. However, the inhibition of S6K1α by β-siRNA showed different results with regard to glycolytic responses, even in the case of β-354 inhibition of de novo glycolytic metabolism through the downregulation of *gk* expression. The overexpression of *pepck* and *g6p* after treatment with β-siRNA showed the opposite trend, as these two rate-limiting enzymes of the gluconeogenic pathway affect the rate of gluconeogenesis [44]. The glycolytic metabolic pathway was significantly enhanced in comparison to the control group. In contrast, Liang et al.’s [19] study results showed that excess dietary leucine levels (2.94%) significantly increased gluconeogenesis by increasing the g6pase and *pepck* mRNA levels through the activation of s6k1. This might be due to the disparate responses of gluconeogenesis, indicating that α and β may perform discrete functions in the S6K1 phenotype. In the present experiment, S6K1α was more intimately involved in the regulation of gluconeogenesis when only S6K1α was inhibited, whereas the inhibition of both S6K1α and S6K1β collectively co-regulated glycolysis. The upregulation of *gs*, in conjunction with the elevated levels of *pepck* and *g6p* after treatment with β-354, indicates that glucose-6-phosphate is ultimately utilized for hepatic glycogen synthesis [45]. With the exception of α-979, the reduced expression of *g6pdh* suggests that glucose is not excessively involved in the pentose phosphate pathway in this fish species, as glucose is not required to produce NADPH for biosynthesis [45,46]. It is noteworthy that treatment with α-979, which promotes glycolysis, also resulted in a considerably higher level of gs expression. It has been demonstrated that the inhibition of S6K activity by the mTOR blocker rapamycin is ineffective in counteracting amino acid-induced inhibition of glucose and palmitate oxidation [14]. This result indicates that this inhibition is independent of mTOR/S6K signaling; that is, mTOR/S6K-mediated competition for mitochondrial substrates, not insulin desensitization, may have special performance characteristics for glucose metabolism.

## 5. Conclusions

In the present experiment, S6K1 played an important role in regulating glucose metabolism in juvenile blunt snout bream. S6K1α was more intimately involved in the regulation of gluconeogenesis when only S6K1α was inhibited, whereas the inhibition of both S6K1α and S6K1β collectively co-regulated glycolysis.

## Figures and Tables

**Table 1:** Primer sequences for real-time PCR analysis.

Genes	Primer Sequence (5′-3′)	Purpose	Accession No.
*s6k1α*-5′race	TGGCGAAAGATGGCAATC	CDS cloning	XP_017572395.1
*s6k1α*-3′race	TACCAGCTGAACAGACAGGACGCC		
*s6k1β*-5′race	AAGGCACGGAGCAGATTC	CDS cloning	XP_016416922.1
*s6k1β*-3′race	GGTGTCTAATGTGGAGCAGATGGA		
*s6k1α*-1489-F	GGGAGUUGCUGGAUAAGAUTT	siRNA knockdown	OR896619.1
*s6k1α*-1489-R	AUCUUAUCCAGCAACUCCCTT		
*s6k1α*-895-F	GGAGCCUAUUCAAACGAAATT	siRNA knockdown	OR896619.1
*s6k1α*-895-R	UUUCGUUUGAAUAGGCUCCTT		
*s6k1α*-979-F	CUACCAUUGACUGGAAUAATT	siRNA knockdown	OR896619.1
*s6k1α*-979-R	UUAUUCCAGUCAAUGGUAGTT		
*s6k1β*-584-F	GAGAACAUCAUGCUCAAUATT	siRNA knockdown	OR896620.1
*s6k1β*-584-R	UAUUGAGCAUGAUGUUCUCTT		
*s6k1β*-681-F	GUGGAACCAUAGAGUACAUTT	siRNA knockdown	OR896620.1
*s6k1β*-681-R	AUGUACUCUAUGGUUCCACTT		
*s6k1β*-354-F	GCAUCCUAGAGGAAGUUAATT	siRNA knockdown	OR896620.1
*s6k1β*-354-R	UUAACUUCCUCUAGGAUGCTT		
*s6k1α*-F	TCGAGGCAAAGGACTTGGTG	RT-PCR	OR896619.1
*s6k1α*-R	CGGGAGGGACGTTCCTATTC		
*s6k1β*-F	GCAGGTCCTCGAGATGCTTT	RT-PCR	OR896620.1
*s6k1β*-R	TGCGAGGTGAACGGGATTTT		
*mtor*-F	ATAGCACACCACAGGGCTTC	RT-PCR	OR902770.1
*mtor*-R	CCAGCAGGTGACCCATAGTG		
*gk*-F	GCTTCCACTGGGATTCACCT	RT-PCR	[19]
*gk*-R	CGACGTTATTGCCTTCAGCG		
*pk*-F	CGAGATTGAGAACGGAGGCA	RT-PCR	[19]
*pk*-R	GTCCTTCTCAGACACTGCGG		
*g6pdh*-F	TGGAGAAACCTTTTGGCCGT	RT-PCR	[19]
*g6pdh*-R	CTGGGTACCAAACGGCTCTT		
*glut2*-F	CGGTGAAACCGAACAGGAGT	RT-PCR	[19]
*glut2*-R	TTCTTTGAGATCGGGCCTGG		
*pepck*-F	TCGCCTGGATGAAGTTCGAC	RT-PCR	[19]
*pepck*-R	GTCTTGGTGGAGGTTCCTGG		
*gs*-F	TTACACGGTCATTGCGTCCA	RT-PCR	[19]
*gs*-R	GACACAGCTCAGTCGGTGAA		
*g6p*-F	TTCAGTGTCACGCTGTTCCT	RT-PCR	[19]
*g6p*-R	TCTGGACTGACGCACCATTT		
*β-actin*-F	TCGTCCACCGCAAATGCTTCTA	RT-PCR	[19]
*β-actin*-R	CCGTCACCTTCACCGTTCCAGT		

**Table 2:** Basic sequence analyses of CDS protein.

Index	S6k1α	S6k1β
molecular formula	C_3736_H_5872_N_1008_O_1096_S_25_	C_2472_H_3883_N_651_O_747_S_26_
molecular weight	83.247 KDa	55.508 KDa
theoretical pI	6.64	5.47
extinction coefficient ^1^	77,240	39,880
instability index ^2^	45.66	42.30
estimated half-life ^3^	30 h/>20 h/>10 h	30 h/>20 h/>10 h
aliphatic index	86.40	77.93
grand average of hydropathicity	−0.343	−0.341

Note: ^1^ extinction coefficient: Abs 0.1% = 0.718, assuming all Cys residues are reduced. ^2^ instability index: both *s6k1α* and *s6k1β* as unstable. ^3^ estimated half-life: 30 h (mammalian reticulocytes, in vivo); >20 h (yeast, in vivo); >10 h (*Escherichia coli*, in vivo).

**Table 3:** Percent identity of S6K1α amino acid sequences between *Megalobrama amblycephala* and 14 other species.

	1	2	3	4	5	6	7	8	9	10	11	12	13	14
1		99.9%	98.8%	98.2%	98.2%	98.0%	97.4%	97.8%	94.6%	78.2%	80.0%	79.2%	79.3%	79.3%
2			98.6%	98.1%	98.1%	98.1%	97.3%	97.7%	94.7%	78.2%	80.0%	79.2%	79.3%	79.3%
3				97.8%	97.7%	97.4%	97.0%	97.7%	94.3%	78.3%	80.0%	79.2%	79.3%	79.3%
4					98.5%	98.2%	96.8%	97.6%	94.5%	78.4%	80.0%	79.2%	79.3%	79.4%
5						99.1%	96.6%	97.7%	94.0%	78.5%	79.9%	79.2%	79.3%	79.4%
6							96.3%	97.4%	94.0%	78.2%	79.6%	78.7%	78.8%	78.8%
7								96.3%	94.0%	77.9%	79.6%	78.8%	79.1%	79.1%
8									94.2%	77.7%	79.6%	78.8%	78.9%	78.7%
9										77.3%	79.3%	78.1%	78.4%	78.0%
10											86.1%	85.8%	85.3%	85.1%
11												92.2%	92.0%	91.8%
12													98.5%	97.6%
13														98.2%
14														

Note: The amino acid sequence identity was calculated with MegAlign of DNAStar. Data were expressed as percentage of amino acid sequence identity. 1. *Megalobrama amblycephala* (GenBank accession no. WPQ74743.1); 2. *Ctenopharyngodon idella* (GenBank accession no. XP_051737588.1); 3. *Pimephales promelas* (GenBank accession no. XP_039540962.1); 4. *Labeo rohita* (GenBank accession no. XP_050954136.1); 5. *Puntigrus tetrazona* (GenBank accession no. XP_043082310.1); 6. *Onychostoma macrolepis* (GenBank accession no. XP_058621277.1); 7. *Myxocyprinus asiaticus* (GenBank accession no. XP_051542966.1); 8. *Carassius auratus* (GenBank accession no. XP_026093793.1); 9. *Danio rerio* (GenBank accession no. XP_009295647.1); 10. *Xenopus tropicalis* (GenBank accession no. NP_001116931.1); 11. *Gallus gallus* (GenBank accession no. AAA21877.1); 12. *Homo sapiens* (GenBank accession no. NP_002944.2); 13. *Mus musculus* (GenBank accession no. AAA50300.1); 14. *Rattus norvegicus* (GenBank accession no. NP_112369.2).

**Table 4:** Percent identity of S6K1β amino acid sequences between *Megalobrama amblycephala* and 10 other species.

	1	2	3	4	5	6	7	8	9	10	11
1		99.8%	98.2%	87.1%	87.6%	84.4%	83.8%	83.2%	89.3%	81.1%	80.9%
2			98.2%	87.3%	87.8%	84.2%	84.0%	83.4%	89.9%	81.1%	80.9%
3				87.5%	87.6%	84.6%	84.2%	83.4%	89.3%	81.3%	81.1%
4					96.0%	82.2%	81.4%	81.0%	85.2%	78.9%	79.3%
5						83.0%	82.4%	82.0%	86.0%	79.5%	79.5%
6							96.9%	96.5%	81.4%	86.8%	86.4%
7								97.6%	80.6%	86.4%	86.0%
8									80.2%	86.0%	85.6%
9										78.3%	78.5%
10											97.6%
11											

Note: The amino acid sequence identity was calculated with MegAlign of DNAStar. Data were expressed as percentage of amino acid sequence identity. 1. *Megalobrama amblycephala* (GenBank accession no. WPQ74744.1); 2. *Anabarilius grahami* (GenBank accession no. ROL54336.1); 3. *Ctenopharyngodon idella* (GenBank accession no. XP_051765238.1); 4. *Xyrauchen texanus* (GenBank accession no. XP_051972942.1); 5. *Myxocyprinus asiaticus* (GenBank accession no. XP_051541960.1); 6. *Lates calcarifer* (GenBank accession no. XP_018547867.1); 7. *Larimichthys crocea* (GenBank accession no. XP_010747297.3); 8. *Sparus aurata* (GenBank accession no. XP_030294597.1); 9. *Danio rerio* (GenBank accession no. XP_021327969.1); 10. *Homo sapiens* (GenBank accession no. NP_001258989.1); 11. *Gallus gallus* (GenBank accession no. NP_001025892.2).

## Data Availability

Data are contained within the article.
